# Role of an automated screening tool for familial hypercholesterolemia in patients with premature coronary artery disease

**DOI:** 10.1016/j.athplu.2022.01.001

**Published:** 2022-01-06

**Authors:** Antti Jokiniitty, Markku Eskola, Tanja Saarela, Heini Huhtala, Saara Metso

**Affiliations:** a Faculty of Medicine and Health Technology, Tampere University, Arvo Ylpön katu 34, 33520, Tampere, Finland; b Department of Internal Medicine, Tampere University Hospital, Elämänaukio 2, 33521, Tampere, Finland; c Department of Clinical Genetics, Kuopio University Hospital, Kuopio, Finland; d Faculty of Social Sciences, Tampere University, Arvo Ylpön katu 34, 33520, Tampere, Finland; e Heart Hospital, Tampere University Hospital, Elämänaukio 1, 33521, Tampere, Finland

**Keywords:** Familial hypercholesterolemia, Coronary artery disease, Screening

## Abstract

**Background and aims:**

To validate an automated screening tool for patients with premature coronary artery disease (CAD) and high total cholesterol or LDL-C levels and assess if it would provide clinicians with additional support in identifying patients with Familial Hypercholesterolemia (FH).

**Methods:**

An IT-based automated screening tool based on coronary angiography data recorded in the KARDIO registry and laboratory values was validated among patients undergone coronary angiography in the Heart Hospital at Tampere University Hospital between 2007 and 2017 fulfilling the criteria of premature CAD (men <55 years and women <60 years) and history of high total cholesterol (>8 mmol/l) or LDL-cholesterol (>5 mmol/l) levels. Electronic health records were retrospectively analyzed to determine if these patients had been diagnosed with FH based on clinical features and whether genetic testing had been conducted.

**Results:**

The automated screening tool identified 0.7% (211/28295) of all patients undergone coronary angiography and revealed history of high cholesterol in 8% (211/2678) of patients with premature CAD during the study period. Fifty-one percent (107/211) of these patients fulfilled the clinical criteria for probable/definite FH based on the Dutch Lipid Clinic Network (DLCN) criteria.

None of the patients had been diagnosed with FH based on clinical criteria before or after diagnosis of CAD. Thirteen percent of patients (n = 14) with probable/definite FH had been tested for genetic mutations of FH before or after CAD, five (36%) of them having a pathogenic FH variant. Two patients were referred to cascade screening.

**Conclusions:**

FH was underdiagnosed among the population studied. An automated screening tool in cardiac care could provide additional support for clinicians in diagnosing patients potentially having FH.

## Highlights

• Familial hypercholesterolemia is underdiagnosed and undertreated in Finland.
• Cardiac care units are important in identification of index patients with Familial Hypercholesterolemia.
• An automated screening tool in cardiac care could provide additional support in diagnosing patients potentially having FH.

## Introduction

Familial hypercholesterolemia (FH) is among the most common autosomal-dominant genetic diseases [1]. Pathogenic mutations in the *LDLR* gene [2], in apolipoprotein B (*APOB*)-100 gene, and *PCSK9* gene [3] can cause reductions in low-density lipoprotein cholesterol (LDL-C) metabolism resulting in elevated LDL-C levels. Untreated severe hypercholesterolemia leads to accelerated atherosclerosis and premature atherosclerotic cardiovascular disease (ASCVD) [4,5].

The estimated prevalence of autosomal dominant FH is 1:311–313 [6,7]. Despite efforts to increase knowledge of the condition it continues to be underdiagnosed and inadequately treated [8]. Globally ∼ 90 – 95% of patients with FH are still undetected [9]. Because coronary artery disease (CAD) is a common and severe complication of FH, cardiac units are crucial in detecting undiagnosed patients. The current guidelines recommend that patients with premature ASCVD, primarily CAD, should be routinely screened for FH [10].

The current ESC guidelines recommend that among CAD patients FH is to be considered among men aged <55 years and women aged <60 years. FH should be diagnosed using clinical criteria and confirmed with DNA analysis when possible. Once the index case is diagnosed, cascade screening, a mechanism for identifying individuals at risk for a genetic condition by a process of systematic family tracing, is recommended [11]. Currently no systematic screening strategies for FH are widely employed in coronary care [12].

The Finns present a genetically isolated population and previously it has been reported that five *LDLR* gene founder mutations account for up to 78% of FH cases and seven founder mutations for up to 90% of cases [13,14]. Based on the prevalence of the Finnish *LDLR* founder mutations in general population, the prevalence of FH in Finland is estimated to be at least 1:600 [15]. The Current Care Guidelines for Dyslipidemia in Finland recommend genetic testing of four FH founder mutations for patients with probable/definite FH based on Dutch Lipid Clinic Network **(**DLCN**)** criteria. Tampere University Hospital is located in the Pirkanmaa region with a population of approximately 520,000. According to the data of the Finnish National Health Insurance (NHI) there are only 284 diagnosed FH patients in our region (prevalence 1:1800).

To improve identification and treatment of FH in our region, we developed an automated screening tool to support clinicians in specialized cardiac care in identifying patients potentially having FH. The aim of this study was to validate the procedure and evaluate the possible advantages of an automated IT-based FH screening tool in cardiac catheterization laboratory compared to current clinician-driven strategy. In addition, our aim was to assess 1) the proportion of patients with premature CAD and probable/definite FH based on DLCN-criteria; 2) the proportion of patients with probable/definite FH who were tested for the four founder *LDLR* mutations as recommended in the Finnish national guidelines; 3) the proportion of genetically confirmed FH cases admitted to the cascade screening protocol.

## Patients and methods

The Heart Hospital in Tampere, Finland is a tertiary center treating all patients in Pirkanmaa Hospital District needing major cardiac or cardiothoracic treatment. In addition to standard hospital electronic health records (EHRs) the Heart Hospital uses the KARDIO registry as a resource planning and quality monitoring application. The KARDIO registry includes prospectively collected detailed information on patients undergoing invasive operations. This includes information on the risk factors for CAD (smoking, diabetes, hypertension, and family history of CAD), patient characteristics (age, gender, anthropometric measures), extent of angiographically verified CAD, and selected treatment modality. The variables recorded in the KARDIO registry are pre-selected before data collection and revised annually with more variables added to the registry [16]. Laboratory values are provided by an accredited laboratory, FimLab and stored in WebFimlab.

Based on the variables recorded in the KARDIO registry and WebFimlab, we created an automated IT-based solution to refer patients with suspected FH to further assessment in the Department of Endocrinology at Tampere University Hospital. The criteria included premature (men <55 years and women <60 years) angiographically verified CAD and total cholesterol (TC) level above 8 mmol/l or LDL-C level above 5 mmol/l at any point in a patient’s laboratory history (WebFimlab) before diagnosis of CAD.

In order to validate the automated screening process and evaluate the possible effects on diagnosis, treatment and cascade screening of patients with FH, we ran our automated screening tool retrospectively, collecting patients exceeding the established thresholds from the KARDIO registry between January 1, 2007 and January 1, 2018. Laboratory values were available for the period January 1, 2000 – January 1, 2018. (Supplement material) After initial screening with the automated screening tool, EHRs were analyzed by a single investigator (AJ) in order to confirm that there were no contradictory data between EHR and the KARDIO registry. In addition, clinical criteria regarding FH, possible genetic testing and cascade screening for FH and reasons for secondary hypercholesterolemia were assessed.

All patients underwent cardiac catheterization during hospitalization and coronary artery disease was defined as > 50% reduction in lumen diameter of any of the three coronary arteries or their main branches. TC and LDL-C levels were obtained from FimLab laboratories. LDL-C was analyzed using the Friedewald equation from January 1, 2000 to March 30, 2017 and using the direct method thereafter since.

We used the DLCN-criteria [17] to identify patients with FH. According to the criteria probable FH was noted for patients scoring 6–8 points, whereas definite FH was noted for patients scoring ≥9 points. In this analysis, patients classified as probable/definite FH were considered to have clinically defined FH [18]. Patients with DLCNC between 3 and 5 were classified as possible FH.

In most cases, we had multiple cholesterol measurements from each patient, and we used the highest TC or LDL-C level assessed before baseline CAD since January 1, 2000. Physical findings of lipid accumulation (tendon xanthomas, arcus cornealis) used in DLCNC were not systematically available and were thus assigned a value of 0. Family history of premature CAD was assessed based on EHRs and information recorded in the KARDIO registry.

Genetic testing for four Finnish FH founder mutations includes FH-Helsinki *LDLR* (g.39215_47749del8535), FH-North-Karelia *LDLR* (c.925_931delCCCATCA, p.(Pro309Lysfs)), FH-Pori *LDLR* (c.1202T > A, p.(Leu401His)), FH-Turku *LDLR* (c.2531G > A, p.(Gly844Asp)).

FH gene panel was conducted by Blueprint Genetics® Hyperlipidemia panel using next-generation sequencing (NGS, including sequencing and deletion/duplication analysis) including *LDLR, APOB, PCSK9, LDLRAP1.* (Supplementary material)

To assess the cardiovascular risk profile of the patients with possible or probable/definite FH, we used the risk factors entered in the KARDIO registry by the cardiologist at the time of angiography. The information was validated with an extensive analysis of EHRs. Diabetes was defined if a patient had a previous diagnosis of diabetes, was receiving treatment for diabetes, or HbA1c level was above 42 mmol/mol. Hypertension was defined as systolic BP > 140 mmHg, diastolic BP > 90mmHg, diagnosis of hypertension, or antihypertensive medication. Smoking was categorized as non-smoker, ex-smoker or current smoker. Obesity was defined as BMI >25 kg/m^2^.

Assessment of secondary causes of hypercholesterolemia was based on EHRs and laboratory values. If there was an apparent secondary reason, *i.e.* nephrotic syndrome or end stage renal disease (ESRD), uncontrolled diabetes mellitus as defined by HbA1c above 70 mmol/l, hypothyroidism (TSH >10mU/l), medications (*i.e.* aripiprazole, anabolic steroids) or cholestasis, patients were excluded from the possible FH group [19]. FH is classically characterized by markedly elevated LDL-C and TC levels. Thus, we set a cutoff point for hypertriglyseridemia at > 10 mmol/l and patients whose fasting TG was above the threshold were excluded. Explanations for high TG included possible familial combined hyperlipidemia, metabolic syndrome and documented excessive alcohol consumption.

The statistical analyses were performed with the SPSS version 26.0 (IBM Corp. Released 2019 Armonk, NY, USA). Absolute numbers and percentages were used to describe categorical data. Categorical variables were analyzed by chi-square test. Quantitative data on age and BMI were given as means and standard deviations (SD) and TC and LDL-C levels as medians and quartiles. Mann-Whitney *U* test was used to assess the difference in continuous variables between the two patient groups. A two-sided *P* value < 0.05 was considered statistically significant.

The study protocol conforms to the ethical guidelines of the 1975 Declaration of Helsinki and the study protocol was approved by the ethics committee of Tampere University Hospital.

## Results

During the study period, 28,295 coronary angiographies (62.3% male) were performed in Tampere Heart Hospital. Mean age at the time of coronary angiography was 65.6 years in males and 69.4 in females. Diabetes was present with 25.5%, hypertension with 64.7% and family history of CAD with 48.2% of all patients. During the time of angiography 17.5% and 20.2% were current or ex-smokers respectively.

**Fig. 1:**
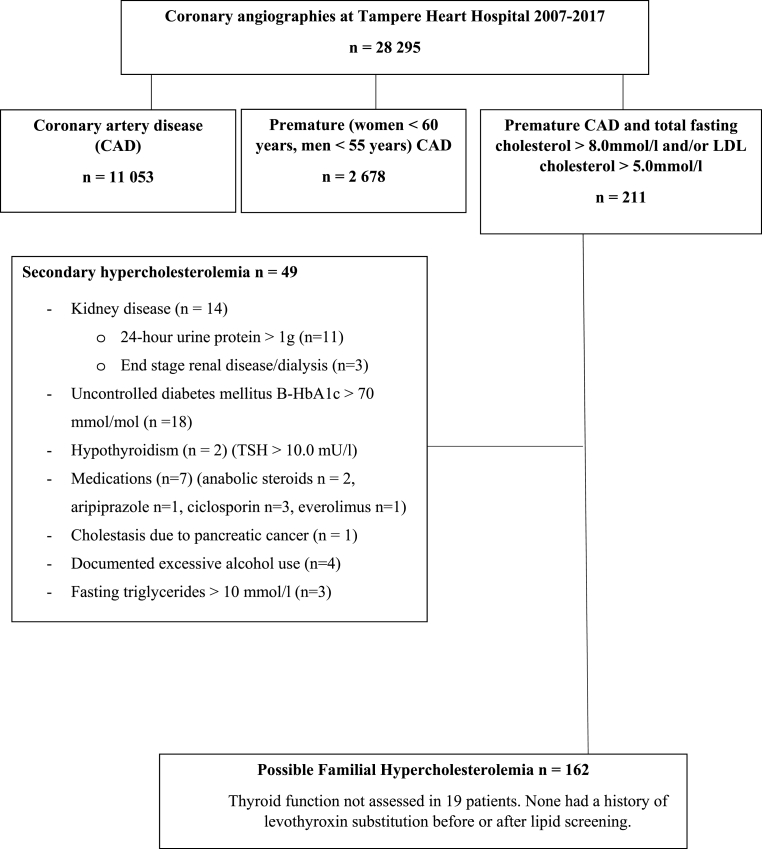
Flow chart of the study.

Premature CAD (men <55 years and women <60 years) was diagnosed in 2,678 patients. Elevated LDL-C (≥5 mmol/l) or TC (≥8 mmol/l) levels were present in 211 patients with premature CAD. Thus, the automated screening tool identified 0.7% (211/28,295) of all patients and 8% (211/2,678) of patients with premature CAD. Forty-nine patients (23%) had an apparent secondary reason for hyperlipidemia and were excluded from further analysis. Thyroid function was not assessed in 19 patients, but none of these patients had a history of levothyroxin substitution before or after lipid screening and were included in the FH analysis. A flow chart of the study is presented in Fig. 1.

Based on the DLCN criteria, 51% (n = 107) of screened patients had probable/definite FH (probable n = 103, definite n = 4). Prevalence of probable/definite FH in patients with premature CAD was 4%. None of these patients had FH diagnosis based on the clinical criteria recorded in their EHRs before or after diagnosis of CAD.

The baseline characteristics of the patients are shown in Table 1. In the probable/definite FH group, 94.4% of the patients had a family history of early CAD while 3.6% of those with possible FH also had this (p=<0.001). The gender distributions in the probable/definite FH and possible FH groups were similar. There were no statistical differences in age at baseline CAD, TC levels, or LDL-C levels between the patients with probable/definite FH and those with possible FH. At the time of referral to coronary angiography, 27.1% of the patients with probable/definite FH and 20.0% of the patients with possible FH were taking lipid lowering drugs (LLD) (p = 0.609). Among those with probable/definite and those with possible FH, 64.4% and 67.3% were current or ex-smokers, respectively (p = 0.162). After diagnosis of CAD the LDL-C goal of <1.8 mmol/l was achieved by 29.9% of the patients with probable/definite FH and 21.8% of those with possible FH (p = 0.273).

**Table 1:** Baseline characteristics of the patients based on DLCN – criteria.

	All		DLCNC ≤5 (n = 55)		DLCNC ≥6 (n = 107)		
	n	%	n	%	n	%	p-value
Gender							0.399
Male	117	72.2	42	76.4	75	70.1	
Female	45	27.8	13	23.6	32	29.9	
Age at baseline CAD, Mean (SD)							0.941
Male	48.5	(4.8)	48.2	(5.7)	48.7	(4.3)	0.982
Female	53.9	(4.8)	55.2	(4.1)	53.4	(5.1)	0.302
TC, Median (Q_1_-Q_3_)	7.4	(7.0–7.8)	7.4	(7.1 – 7.7)	7.4	(6.9 – 7.9)	0.910
LDL-C, Median (Q_1_-Q_3_)	5.4	(5.1–5.8)	5.4	(5.1 – 5.7)	5.4	(5.2 – 5.9)	0.088
LDL-C, mmol/l							0.025
≤ 6.4	145	89.5	54	98.2	91	85.0	
6.5 – 8.4	14	8.6	1	1.8	13	12.1	
≥ 8.5	3	1.9	0	0	3	2.9	
Family history of early CAD	103	63.6	2	3.6	101	94.4	< 0.001
Hypertension	115	71.0	32	58.2	83	77.6	0.010
Diabetes	27	16.6	9	16.4	18	16.8	0.941
Smoking at baseline CAD							0.162
Non-smoker	51	31.5	18	32.7	33	30.8	
Ex-smoker	47	29.0	11	20.0	36	33.6	
Current smoker	64	39.5	26	47.3	38	35.5	
BMI, Mean (SD)	30.0	(5.9)	29.6	(6.7)	30.2	(5.5)	0.306
LLD at baseline CAD							0.609
Yes	40	24.7	11	20.0	29	27.1	
Prescribed but not in use	19	11.7	7	12.7	12	11.2	
LDL-C ≤ 1.8 mmol/l achieved after CAD	44	27.2	12	21.8	32	29.9	0.273

DLCNC, Dutch lipid clinic network criteria; CAD, coronary artery disease; TC, total cholesterol; LDL-C, low-density lipoprotein cholesterol; BMI, body mass index; LLD, lipid lowering drug.

**Fig. 2:**
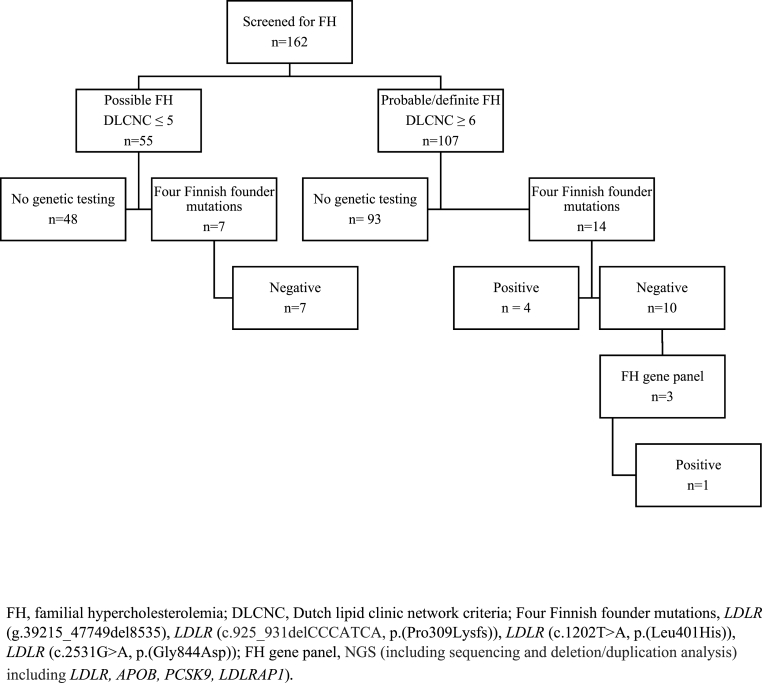
Flow chart of genetic testing FH, familial hypercholesterolemia; DLCNC, Dutch lipid clinic network criteria; Four Finnish founder mutations, *LDLR* (g.39215_47749del8535), *LDLR* (c.925_931delCCCATCA, p.(Pro309Lysfs)), *LDLR* (c.1202T > A, p.(Leu401His)), *LDLR* (c.2531G > A, p.(Gly844Asp)); FH gene panel, NGS (including sequencing and deletion/duplication analysis) including *LDLR, APOB, PCSK9, LDLRAP1*)*.*

In the screened population, 21 patients were studied for genetic mutations of FH, 14 in the probable/definite FH group and seven in the possible FH group, as illustrated in Fig. 2. The baseline characteristics based on genetic testing are shown in Table 2. Patients referred to genetic testing were more likely to be male than female (p = 0.049) and had higher LDL-C levels than those who were not referred to genetic testing (5.6 mmol/l vs 5.3 mmol/l, p = 0.004). Three patients were further studied with FH gene panels as a follow-up. Five pathogenic FH variants were discovered (36% of the patients tested). Ninety-three (87%) patients with probable/definite FH were not tested for genetic mutations of FH.

**Table 2:** Baseline characteristics of patients based on genetic testing.

	No genetic testing		Genetic testing		
	n	%	n	%	p-value
Gender					0.098
Male	105	73.9	12	60.0	
Female	36	25.2	9	42.9	
Age at baseline CAD, Mean (SD)					0.105
Male	48.8	(4.7)	45.9	(5.4)	0.049
Female	54.5	(4.4)	51.6	(6.0)	0.172
TC, Median (Q_1_-Q_3_)	7.4	(7.0 – 7.8)	7.3	(7.1 – 9.4)	0.174
LDL-C, Median (Q_1_-Q_3_)	5.3	(5.1 – 5.8)	5.6	(5.4 – 7.0)	0.004
LDL-C, mmol/l					0.001
< 6.4	128	90.8	14	66.7	
6.4 – 8.4	13	9.2	3	14.3	
> 8.4	0	0.0	4	19.0	
DLCNC – score					0.006
≤ 5	48	34.0	7	33.3	
6 – 8	93	66.0	10	47.6	
≥ 9	0	0	4	19.0	
Family history of early CAD	90	63.8	13	61.9	0.864
Hypertension	99	70.2	16	76.2	0.573
Diabetes	22	15.6	5	23.8	0.346
Smoking					0.752
Non-smoker	43	30.5	8	38.1	
Ex-smoker	41	29.1	6	28.6	
Current smoker	57	40.4	7	33.3	
BMI, Mean (SD)	30.0	6.0	29.9	5.6	0.991
LLD at baseline CAD	33	23.4	7	33.3	0.247
LLD prescribed but not in use	15	10.6	4	19.0	
LDL-C ≤ 1.8 mmol/l achieved after CAD	38	27.0	6	28.6	0.876

DLCNC, Dutch lipid clinic network criteria; CAD, coronary artery disease; TC, total cholesterol; LDL-C, low-density lipoprotein cholesterol; BMI, body mass index; LLD, lipid lowering drug.

Two patients with a confirmed mutation were referred to genetic counseling for cascade screening.

## Discussion

This study demonstrates that using an automated screening tool in cardiac care based on easily available variables will enhance the identification of patients with FH. During the study period in a population of 520,000 and from 28,295 coronary angiographies the automatic screening tool identified 107 patients fulfilling the clinical criteria of FH (1:2 of the patients screened). There is an apparent lack of awareness regarding clinical diagnostic criteria of FH in our area since none of the patients were diagnosed with FH based on clinical features. In addition, genetic testing of FH has been seldom used in our area. Although patients with markedly elevated LDL-C levels are more likely to be tested for founder mutations, majority of patients with probable FH were not studied for genetic mutations of FH as recommended in Current Care Guidelines and one third of the testing was directed to patients with lower pre-test probability (possible FH) when the decision for testing was based solely on clinicians’ awareness. Automated screening tool would have increased the number of patients referred to genetic testing from 14 to 107 patients in the probable/definite FH group.

Early diagnosis of FH is vital in order to initiate adequate treatment and follow-up procedures for the affected patients before serious adverse events occur. In order to achieve this, several different strategies have been studied. Universal testing has yielded only modest results [20]. Several different EHR-derived FH classifiers have been developed worldwide in order to identify candidate patients for further FH screening. Machine learning guided strategies as well as predictive modeling tools can results in the effective identification of the highest risk patients for enhanced management strategies [[21], [22], [23]]. At the moment, there are no solely EHR based diagnostic tools for FH available in Finland.

There is an ongoing discussion about the value of routine genetic testing of FH patients. The financial burden and lack of availability of genetic testing present the largest hurdles. In a population like that of Finland, with founder mutations well documented and testing readily available, the ideal goal would be to diagnose FH patients on the basis of genotype. Confirming the molecular genetic diagnosis is important in establishing cascade screening because, due to the dominant inheritance pattern of FH, this has been proven to be highly effective [24]. It is estimated that in 60–80% of definitive FH and in 20–30% of possible FH cases a genetic mutation can be found [25]. However, identifying the proband remains a challenge.

In our study population, the prevalence of FH in patients with premature CAD was 1:25 compared to an estimated prevalence of 1:600 in general population. The markedly higher prevalence of FH in patients with premature CAD or ACS has been documented elsewhere [7]. Farnier et al. determined that using DLCN-criteria FH was probable/definite in 2.1% of patients referred to hospital for ACS [26]. Dyrbús et al. reported a prevalence of 1.6% in all ACS patients [27]. In both studies, ACS patients with probable/definite FH were significantly younger than non-FH patients at the time of ACS by almost 20 years. Focusing on patients with premature CAD and using genetic testing, Amor-Salamanca et al. reported an 8.7% prevalence of FH among patients with ACS under 65 years and LDL-C above 4.1 mmol/l [28]. In a recent meta-analysis the pooled prevalence of FH in ACS for patients aged <60 years was 7.3% (95% CI, 5.3–10.0) and increased to 13.7% (95% CI, 8.2–22.1) for those aged ≤45 years [29].

Patients with premature CAD and elevated cholesterol levels present a population where genetic testing is likely to offer the greatest clinical advantages. Identifying the FH index patients enables genetic counseling and subsequent screening of family members to optimize primary prevention and possibly prevent serious atherosclerotic cardiovascular complications.

It is moreover noteworthy that in both the probable/definite and possible FH groups smoking (current/ex) was markedly more common than in general population as approximately 15% of all Finnish people aged 20–64 smoked daily in 2016 [30]. LLD was used as primary prevention by only 25% (40/162) patients in total. Only 27% of the patients with premature CAD with elevated TC and LDL-C reached an LDL-C level of <1.8 mmol/l during follow-up, which is comparable to that reported in other studies [31].

The screened patients with premature CAD in addition to having either monogenic or complex hyperlipidemia also had several other risk factors for ASCVD. Better identification of these patients and enhancing primary and secondary prevention in this population is required.

The automated screening tool would have created 20–30 referrals per year from the Heart Hospital to the Endocrine Clinic for clinical evaluation and genetic testing of the FH candidates. The screening tool markedly narrows down the number of patients for enhanced analysis regarding FH and genetic testing.

In the future, to avoid unnecessary referrals due to the relatively large number of patients with apparent secondary hypercholesterolemia, the screening tool could include values to exclude patients with e.g. ESRD, keeping in mind that IT-based solutions are structured solely to support clinicians and thus patients with suspected FH should be thoroughly evaluated in a special clinic.

The diagnosis of FH in this study is based on clinical criteria, but the patient cohort collected with the automated solution creates a unique opportunity to explore possible genotypic variants of FH in our local population with a high pre-test probability.

After the study, with improved screening the ratio of diagnosed FH patients calculated from the expected total number of FH individuals in the region would increase. Establishing a genetic diagnosis for patients with FH and further cascade screening could increase the prevalence of FH in our region even further. Yet the prevalence of FH seems to remain markedly lower than the estimated 1:311–313 worldwide and 1:600 in Finnish population.

More studies are needed to optimize threshold values in the automated screening tool and to assess the prevalence of FH in general population and in patients with premature CAD in our area.

## Financial support

This study was financially supported by the Competitive State Research Financing of the Expert Responsibility area of 10.13039/501100010600Tampere University Hospital and 10.13039/501100010600Tampere University Hospital Support Foundation, 10.13039/501100010600Tampere University Hospital. This study was financially partly supported by an unrestricted grant from 10.13039/100002429Amgen given to the Heart Hospital Tampere University Hospital.

## Author contributions

A.J. made substantial contributions to the design of the work, the acquisition, analysis, and interpretation of the data, drafting the manuscript, and revising it. S.M., M.E., and T.S. made substantial contributions to the design of the work, the interpretation of the data and revising the manuscript. H.H. made substantial contributions to the analysis and interpretation of the data. All authors approved the submitted version and agreed to be personally accountable for the author's own contributions and to ensure that questions related to the accuracy or integrity of any part of the work, even those in which the author was not personally involved, will be appropriately investigated, resolved, and the resolution documented in the literature.

## Declaration of competing interest

The authors declare that they have no known competing financial interests or personal relationships that could have appeared to influence the work reported in this paper.
