# Supplementary material for: Role of an automated screening tool for familial hypercholesterolemia in patients with premature coronary artery disease
Source: Atheroscler Plus. 2022 Jan 6;48:1–7. doi: 10.1016/j.athplu.2022.01.001 (PMC9833226; doi:10.1016/j.athplu.2022.01.001)
Supplement: Multimedia component 1 [file mmc1.docx]

**Supplementary Material**

Manuscript: **Role of an automated screening tool for familial hypercholesterolemia in patients with premature coronary artery disease.** Jokiniitty et al.

1. **Details of data collection**

Clinical cardiovascular phenotype data is continuously recorded during hospitalization of patients undergoing invasive procedures and treated at the critical care unit and/or at the cardiac ward by cardiologists (KARDIO –registry). KARDIO-registry data comprises detailed information on the extent of angiographically verified coronary artery disease, patient status upon admission and during treatment, selected treatment modality, treatment success and patient’s prevalent conditions as determined by treating physicians. Variables recorded in the KARDIO-registry are pre-selected before implementation of data collection based on the expected clinical significance of the variables in predicting serious adverse events. The data collection protocol is under continuous revision with more variables added to the registry annually. (Supplementary Table 1)

TC and LDL-C levels are extracted directly from hospitals EHRs (WebFimlab provided by FimLab) and values from 1.1.2000 onwards are available for each patient. If a patient is taking lipid lowering drug, pre-treatment values are not systematically calculated. However, the screening tool searches for all TC/LDL-C levels from 1.1.2000 onwards and selects patients according to their highest cholesterol values. Thus, pre-treatment measurements are probably available and used in most cases. The screening tool is unable to differentiate the reasons for secondary hypercholesterolemia (medications, comorbidities, excessive alcohol consumption etc.) from primary hypercholesterolemia.

The main goal of this study was to evaluate the possible effect of an automated screening tool on the diagnosis, treatment, and cascade screening of patients with possible FH compared to the current protocol, which is based strictly on treating-clinicians knowledge. Using the values selected for the screening tool we collected patients fulfilling the criteria between 1.1.2007 and 1.1.2018 from the KARDIO-registry. As patients were not systematically evaluated for secondary hypercholesterolemia or FH during treatment, we did this retrospectively based on the KARDIO-registry and data from national and hospital electronic health records. (Supplementary - Table 1)

Supplementary Figure 1 – Dataflow

**Electronic health records of Tampere University Hospital (AJ):**

Validation of the patients identified with the automated tool for clinical FH

Assessment of secondary reasons for hypercholesterolemia

Retrospective analysis of EHR´s on the diagnosis, genetic testing, and cascade screening of FH and treatment of hypercholesterolemia.

**WebFimlab**

Total cholesterol and LDL-cholesterol since 1.1.2000

**Screening tool for FH**

Coronary artery disease: percutaneous coronary intervention (PCI), coronary artery bypass operation, angiography finding of >50 % stenosis in 1, 2 or 3 vessels or left main artery, CAD-diagnose (I20-I25).

AND

Age in men < 55 years and women < 60 years

AND

total cholesterol (> 8 mmol/l) or LDL-cholesterol (> 5 mmol/l) levels

**KARDIO-Registry**

Patient characteristics: age, gender

Coronary artery disease: percutaneous coronary intervention (PCI), coronary artery bypass operation, angiography finding of >50 % stenosis in 1, 2 or 3 vessels or left main artery, CAD-diagnose (I20-I25). Selected treatment modality.

Risk factors for CAD: Smoking, diabetes, hypertension, family history of CAD

Supplementary Table 1 – Data collection sources

| Variable | Definition | Sources and variable construction |
| --- | --- | --- |
| **Basic characteristics** |  |  |
| Age | Patient´s age at the time of coronary angiography | Extracted directly from hospitals EHRs to KARDIO-registry |
| Sex (female) | Gender | Extracted directly from hospitals EHRs to KARDIO-registry |
| Operation year | The year in which the patient was diagnosed with CAD by coronary angiography | Recorded directly in the KARDIO-registry by a treating physician |
| #3 |  |  |
| **Comorbidities** |  |  |
| Diabetes | Prevalent diabetes (type I or type II) | Recorded directly to KARDIO-registry by a treating physician |
| Type I diabetes | Prevalent type I diabetes | Recorded directly to KARDIO-registry by a treating physician |
| Type II diabetes | Prevalent type II diabetes | Recorded directly to KARDIO-registry by a treating physician |
| Stroke | Stroke before baseline CAD | Recorded directly to KARDIO-registry by a treating physician |
| Hypertension | Pre-existing hypertension (any) or diagnosed hypertension during hospitalization at baseline CAD | Recorded directly to KARDIO-registry by a treating physician |
| Peripheral artery disease (PAD) | Pre-existing clinically significant PAD (any) before baseline CAD | Recorded directly to KARDIO-registry by a treating physician |
| Previous CKD | Chronic Kidney Disease (24-hour urine protein > 1g, ESRD/dialysis) before baseline CAD | Recorded directly to KARDIO-registry by a treating physician |
| #7 |  |  |
| **Other Risk Factors** |  |  |
| Family history of premature CAD/vascular disease | First-degree relative with known premature (men < 55 years, women < 60 years) coronary or vascular disease | Recorded directly to KARDIO-registry by a treating physician |
| Smoking | Current smoker, ex-smoker, non-smoker at baseline CAD | Recorded directly to KARDIO-registry by a treating physician |
| #2 |  |  |
| **Angiographic findings/events** |  |  |
| CAD severity | Ordinal scale based on visual estimate: 0=no stenosis, 1=<50% stenosis, 2=one vessel with ≥50% stenosis, 3=two vessels with ≥50% stenosis, 4=three vessels with ≥50% stenosis. | Recorded directly to KARDIO-registry by a treating physician |
| Number of stenosed segments | Ordinal scale based on the total number of epicardical segments with ≥50% stenosis. | Recorded directly to KARDIO-registry by a treating physician |
| Significant stenosis (yes/no) | Binary variable: 0=no epicardial arteries with ≥50% stenosis, 1=one or more epicardial artery with ≥50% stenosis. | Recorded directly to KARDIO-registry by a treating physician |
| 1-vessel CAD | 1-vessel CAD (yes/no) | Recorded directly to KARDIO-registry by a treating physician |
| 2-vessel CAD | 2-vessel CAD (yes/no) | Recorded directly to KARDIO-registry by a treating physician |
| 3-vessel CAD | 3-vessel CAD (yes/no) | Recorded directly to KARDIO-registry by a treating physician |
| Stenosed LAD | ≥50% stenosis in LAD (yes/no) | Recorded directly to KARDIO-registry by a treating physician |
| Stenosed LXC | ≥50% stenosis in LXC (yes/no) | Recorded directly to KARDIO-registry by a treating physician |
| Stenosed RCA | ≥50% stenosis in RCA (yes/no) | Recorded directly to KARDIO-registry by a treating physician |
| Angio puncture site (femoral) | Access site for coronary angiography | Recorded directly to KARDIO-registry by a treating physician |
| Left main branch involvement | Significant obstructing stenosis in left main branch. | Recorded directly to KARDIO-registry by a treating physician |
| TIMI at angiography | Coronary artery flow of the culprit artery recorded using TIMI grade | Recorded directly to KARDIO-registry by a treating physician |
| TIMI after PCI | Coronary artery flow of the culprit artery recorded using TIMI grade. If no culprit artery was identified or in the presence of stable chronic total obstruction this scale is coded as 0. | Recorded directly to KARDIO-registry by a treating physician |
| Circulation Dominance | Right coronary artery dominance in angiography | Recorded directly to KARDIO-registry by a treating physician |
| Complications in PCI | Any type of complication in during PCI | Recorded directly to KARDIO-registry by a treating physician |
| Complications in angiography | Any type of complication in during angiography | Recorded directly to KARDIO-registry by a treating physician |
| #17 |  |  |
| **Clinical chemistry – automated FH screening tool** |  |  |
| Max Total Cholesterol (TC) | Maximum value of TC concentration before baseline CAD. | Extracted directly from hospitals EHRs (WebFimlab provided by FimLab) with automated FH screening tool |
| Max Low-density lipoprotein cholesterol (LDL-C) | Maximum value of LDL-C concentration before baseline CAD.  (LDL-C was analyzed using the Friedewald equation from 1^st^ Jan 2000 until 30^th^ March 2017 and using the direct method thereafter) | Extracted directly from hospitals EHRs (WebFimlab provided by FimLab) with automated FH screening tool |
| #2 |  |  |
| **Clinical Chemistry 2** |  |  |
| Min Low-density lipoprotein cholesterol (LDL-C) | Minimum value of LDL-C concentration after baseline CAD.  (LDL-C was analyzed using the Friedewald equation from 1^st^ Jan 2000 until 30^th^ March 2017 and using the direct method thereafter) | Extracted directly from hospitals EHRs (WebFimlab provided by FimLab) |
| Max High-density lipoprotein cholesterol (HDL-c) | Maximum value of HDL-c concentration before baseline CAD. | Extracted directly from hospitals EHRs (WebFimlab provided by FimLab) |
| Triglycerides (Trigly) | Triglyceride concentration at the time of highest LDL-C/TC measurement | Extracted directly from hospitals EHRs (WebFimlab provided by FimLab) |
| Creatinine | Creatinine concentration at the time of highest LDL-C/TC measurement | Extracted directly from hospitals EHRs (WebFimlab provided by FimLab) |
| Glucose | Glucose concentration at the time of highest LDL-C/TC measurement | Extracted directly from hospitals EHRs (WebFimlab provided by FimLab) |
| Hemoglobin A1c | Hemoglobin A1c concentration at the time of highest LDL-C/TC measurement | Extracted directly from hospitals EHRs (WebFimlab provided by FimLab) |
| 24-hour urine protein | 24-hour urine protein at the time of highest LDL-C/TC measurement | Extracted directly from hospitals EHRs (WebFimlab provided by FimLab) |
| Thyroid-Stimulating Hormone | Thyroid-Stimulating hormone concentration at the time of highest LDL-C/TC measurement | Extracted directly from hospitals EHRs (WebFimlab provided by FimLab) |
| Tetraiodothyronine | Tetraiodothyronine concentration at the time of highest LDL-C/TC measurement | Extracted directly from hospitals EHRs (WebFimlab provided by FimLab) |
| #9 |  |  |
| **Genetic testing** |  |  |
| Finnish founder mutations | Four Finnish founder mutations – see Supplementary Table 3a | Extracted directly from hospitals EHRs (WebFimlab provided by FimLab) |
| FH Gene panel | FH-Gene panel (Blueprint Genetics) – see Supplementary Table 3b and 3c | Extracted directly from hospitals EHRs (WebFimlab provided by FimLab) |
| #2 |  |  |
| **Comorbidities – secondary hypercholesterolemia** |  |  |
| Alcohol | Documented excessive use of alcohol | Value added by investigator (AJ) from EHRs |
| Cholestatic disease | Cholestatic disease at the time of highest LDL-C/TC measurement | Value added by investigator (AJ) from EHRs |
| #2 |  |  |
| **Medications** |  |  |
| Lipid lowering drugs | 1. Lipid lowering drug (any) at baseline CAD 2. Lipid lowering drug (any)prescribed previously but not in use at baseline CAD 3. No lipid lowering drugs (any) before CAD | Value added by investigator (AJ) from EHRs |
| Glucose lowering drugs | Glucose lowering drug (any) before baseline CAD | Value added by investigator (AJ) from EHRs |
| Antihypertensive drugs | Antihypertensive drug (any) before baseline CAD | Value added by investigator (AJ) from EHRs |
| Anabolic steroids | Anabolic steroids (any) at the time of highest LDL-C/TC measurement | Value added by investigator (AJ) from EHRs |
| Antipsychotic drugs | Antipsychotic drug (any) at the time of highest LDL-C/TC measurement | Value added by investigator (AJ) from EHRs |
| Immunosuppressive drugs | Immunosuppressive drugs (any) at the time of highest LDL-C/TC measurement | Value added by investigator (AJ) from EHRs |
| #6 |  |  |

1. **Dutch Lipid Clinic Network diagnostic criteria for Familial Hypercholesterolemia**

To establish clinical-FH diagnosis we used the DLCN – criteria where score six or above was classified as FH. (Supplementary Table 2).

Supplementary Table 2. Dutch Lipid Clinic Network diagnostic criteria for Familial Hypercholesterolemia ^1-2^

| Criteria | Points |
| --- | --- |
| **Family history** |  |
| First-degree relative with known premature* coronary and vascular disease, OR 1 First-degree relative with known LDL-C level above the 95th percentile | 1 |
| First-degree relative with tendinous xanthomata and/or arcus cornealis, OR 2 Children aged less than 18 years with LDL-C level above the 95th percentile | 2 |
| **Clinical history** |  |
| Patient with premature* coronary artery disease | 2 |
| Patient with premature* cerebral or peripheral vascular disease | 1 |
| **Physical examination** |  |
| Tendinous xanthomata | 6 |
| Arcus cornealis prior to age 45 years | 4 |
| **Cholesterol levels mmol/liter (mg/dl)** |  |
| LDL-C ≥ 8.5 (330) | 8 |
| LDL-C 6.5–8.4 (250 – 329) | 5 |
| LDL-C 5.0–6.4 (190 – 249) | 3 |
| LDL-C 4.0–4.9 (155 – 189) | 1 |
| **DNA analysis**** |  |
| Functional mutation in the LDLR, apo B or PCSK9 gene | 8 |
| **Diagnosis (diagnosis is based on the total number of points obtained)** |  |
| Definite Familial Hypercholesterolemia | ≥9 |
| Probable Familial Hypercholesterolemia | 6 – 8 |
| Possible Familial Hypercholesterolemia | 3 – 5 |
| Unlikely Familial Hypercholesterolemia | <3 |

* Premature = < 55 years in men; < 60 years in women

LDL-C = low density lipoprotein cholesterol; FH, familial hypercholesterolemia.

LDLR = low density lipoprotein receptor

Apo B = apolipoprotein B

PCSK9 = Proprotein convertase subtilisin/kexin type 9

1. **Genetic testing**

Genetic analysis for four Finnish founder mutations of the *LDLR* gene (FH-helsinki, FH-North-Karelia, FH-Pori, FH-Turku) was conducted by FimLab. Samples were extracted from peripheral blood (min. 1ml) in an EDTA tube. Quantitative polymerase chain reaction with TaqMan® SNP Genotyping assay, Applied Biosystems® with specific primers and probes for each founder mutation was conducted with LightCycler 480 II, Roche® (Supplementary table 3a)

FH gene panel was conducted by Blueprint Genetics® Hyperlipidemia panel using next-generation sequencing (NGS including sequencing and deletion/duplication analysis). Blueprint Genetics Laboratory located in Finland is a CLIA-certified laboratory and accredited by the College of American Pathologists and by FINAS Finnish Accreditation Service. Samples were extracted from peripheral blood (min. 1ml) in an EDTA tube. The target region for each gene includes coding exons and ±20 base pairs from the exon-intron boundary. In addition, the panel includes non-coding and regulatory variants listed in Supplementary Table 3c. (Panel content is described in Supplementary Tables 3b and 3c)

Supplementary Table 3a – Finnish “founder” mutations

| Gene | Genomic location HG19 | HGVS | RefSeq | RS-number | Name |
| --- | --- | --- | --- | --- | --- |
| *LDLR* | chr19: 11123590-11132124 | g.39215_47749del8535 | NG_009060.1 |  | FH-Helsinki |
| *LDLR* | chr19:11107498-11107505 | c.925_931delCCCATCA  p.(Pro309Lysfs) | NM_000527.5 | 387906304 | FH-North-Karelia |
| *LDLR* | chr19:11113293 | c.1202T>A  p.(Leu401His) | NM_000527.5 | 121908038 | FH-Pori |
| *LDLR* | chr19:11129654 | c.2531G>A  p.(Gly844Asp) | NM_000527.5 | 121908037 | FH-Turku |

## Supplementary Table 3b - Panel Content: Genes in the Hyperlipidemia Panel (Blueprint genetics®)

| HGNC approved gene symbol |
| --- |
| *ABCA1* |
| *ABCG5* |
| *ABCG8* |
| *ALMS1* |
| *APOA1* |
| *APOA5* |
| *APOB* |
| *APOC2* |
| *APOC3* |
| *APOE* |
| *CREB3L3* |
| *GPIHBP1* |
| *LDLR* |
| *LDLRAP1* |
| *LIPA* |
| *LMF1* |
| *LPL* |
| *PCSK9* |

#### Supplementary Table 3c - Non-coding variants covered by Hyperlipidemia Panel (Blueprint Genetics®)

| Gene | Genomic location HG19 | HGVS | RefSeq | RS-number |
| --- | --- | --- | --- | --- |
| *ABCA1* | Chr9:107549295 | c.6205-39delT | NM_005502.3 | rs572405590 |
| *ABCA1* | Chr9:107567035 | c.4465-34A>G | NM_005502.3 |  |
| *ABCA1* | Chr9:107571856 | c.4176-11T>G | NM_005502.3 |  |
| *ABCA1* | Chr9:107599404 | c.1195-27G>A | NM_005502.3 | rs200563809 |
| *ABCA1* | Chr9:107690213 | c.-93+2dupT | NM_005502.3 |  |
| *APOA1* | Chr11:116708299 | c.-21+22G>A | NM_000039.1 |  |
| *APOA1* | Chr11:116708365 | c.-65A>C | NM_000039.1 |  |
| *APOC3* | Chr11:116701284 | c.-13-2A>C | NM_000040.1 |  |
| *LDLR* | Chr19:11199939 |  | NM_000527.4 |  |
| *LDLR* | Chr19:11199958 | c.-267A>G | NM_000527.4 |  |
| *LDLR* | Chr19:11199997 | c.-228G>C | NM_000527.4 | rs376713337 |
| *LDLR* | Chr19:11200000 |  | NM_000527.4 |  |
| *LDLR* | Chr19:11200019 | c.-206C>T | NM_000527.4 | rs549995837 |
| *LDLR* | Chr19:11200031 |  | NM_000527.4 | rs1270618112 |
| *LDLR* | Chr19:11200032 |  | NM_000527.4 | rs879254362 |
| *LDLR* | Chr19:11200032 |  | NM_000527.4 |  |
| *LDLR* | Chr19:11200034 | c.-191C>A | NM_000527.4 |  |
| *LDLR* | Chr19:11200037 | c.-188C>T | NM_000527.4 |  |
| *LDLR* | Chr19:11200038 | c.-185_-183delCTT | NM_000527.4 |  |
| *LDLR* | Chr19:11200053 | c.-172G>A | NM_000527.4 |  |
| *LDLR* | Chr19:11200057 | c.-168A>G | NM_000527.4 |  |
| *LDLR* | Chr19:11200062 | c.-163T>C | NM_000527.4 |  |
| *LDLR* | Chr19:11200064 | c.-161A>C | NM_000527.4 |  |
| *LDLR* | Chr19:11200069 | c.-156C>T | NM_000527.4 |  |
| *LDLR* | Chr19:11200069 | c.-155_-154delACinsTTCTGCAAACTCCT | NM_000527.4 |  |
| *LDLR* | Chr19:11200069 | c.-155_-150delACCCCA | NM_000527.4 |  |
| *LDLR* | Chr19:11200070 | c.-155_-154delACinsTTCTGCAAACTCCT | NM_000527.4 | rs879254365 |
| *LDLR* | Chr19:11200070 | c.-155_-150delACCCCAinsTT | NM_000527.4 |  |
| *LDLR* | Chr19:11200071 | c.-154C>T | NM_000527.4 |  |
| *LDLR* | Chr19:11200072 | c.-153C>T | NM_000527.4 |  |
| *LDLR* | Chr19:11200073 | c.-152C>T | NM_000527.4 |  |
| *LDLR* | Chr19:11200074 | c.-151C>G | NM_000527.4 |  |
| *LDLR* | Chr19:11200075 | c.-150A>G | NM_000527.4 |  |
| *LDLR* | Chr19:11200076 | c.-149C>A | NM_000527.4 |  |
| *LDLR* | Chr19:11200079 | c.-146C>A | NM_000527.4 |  |
| *LDLR* | Chr19:11200083 | c.-142C>G/T | NM_000527.4 |  |
| *LDLR* | Chr19:11200084 | c.-139_-130delCTCCCCCTGC | NM_000527.4 |  |
| *LDLR* | Chr19:11200085 | c.-140C>A/G/T | NM_000527.4 | rs875989887 |
| *LDLR* | Chr19:11200086 | c.-139C>A/G | NM_000527.4 |  |
| *LDLR* | Chr19:11200086 | c.-138delT | NM_000527.4 | rs387906307 |
| *LDLR* | Chr19:11200087 | c.-138T>C | NM_000527.4 |  |
| *LDLR* | Chr19:11200088 | c.-137C>T | NM_000527.4 |  |
| *LDLR* | Chr19:11200089 | c.-136C>G/T | NM_000527.4 |  |
| *LDLR* | Chr19:11200089 | c.-136C>G | NM_000527.4 | rs879254374 |
| *LDLR* | Chr19:11200089 | c.-136C>T | NM_000527.4 |  |
| *LDLR* | Chr19:11200090 | c.-135C>G | NM_000527.4 |  |
| *LDLR* | Chr19:11200091 | c.-134C>T | NM_000527.4 |  |
| *LDLR* | Chr19:11200098 | c.-124dupA | NM_000527.4 |  |
| *LDLR* | Chr19:11200105 | c.-120C>T | NM_000527.4 | rs875989886 |
| *LDLR* | Chr19:11200124 | c.-101T>C | NM_000527.4 | rs747068848 |
| *LDLR* | Chr19:11200126 | c.-99A>G | NM_000527.4 |  |
| *LDLR* | Chr19:11200127 | c.-98C>T | NM_000527.4 |  |
| *LDLR* | Chr19:11200202 | c.-23A>C | NM_000527.4 | rs763282380 |
| *LDLR* | Chr19:11200202 | c.-22delC | NM_000527.4 | rs879254379 |
| *LDLR* | Chr19:11200211 | c.-14C>A | NM_000527.4 |  |
| *LDLR* | Chr19:11218203 | c.940+14delC | NM_000527.4 | rs879254730 |
| *LDLR* | Chr19:11221315 | c.941-13T>A | NM_000527.4 |  |
| *LDLR* | Chr19:11224179 | c.1359-31_1359-23delGCGCTGATGinsCGGCT | NM_000527.4 |  |
| *LDLR* | Chr19:11224186 | c.1359-25A>G | NM_000527.4 |  |
| *LDLR* | Chr19:11227685 | c.1845+11C>G | NM_000527.4 |  |
| *LDLR* | Chr19:11227689 | c.1845+15C>A | NM_000527.4 |  |
| *LDLR* | Chr19:11231284 | c.2140+86C>G | NM_000527.4 |  |
| *LDLR* | Chr19:11231301 | c.2140+103G>T | NM_000527.4 |  |
| *LDLR* | Chr19:11242035 | c.*43G>A | NM_000527.4 | rs879254527 |
| *LDLRAP1* | Chr1:25870164 | c.-17_-12dupGGCGGC | NM_015627.2 |  |
| *LDLRAP1* | Chr1:25891056 | c.748-608G>A | NM_015627.2 |  |
| *LPL* | Chr8:19796711 | c.-241G>C | NM_000237.2 | rs540525285 |
| *LPL* | Chr8:19796725 | c.-227T>C | NM_000237.2 |  |
| *PCSK9* | Chr1:55505180 | c.-331C>A | NM_174936.3 | rs778796405 |

References:

1. Nordestgaard BG, Chapman MJ, Humphries SE, et al. Familial hypercholesterolaemia is underdiagnosed and undertreated in the general population: guidance for clinicians to prevent coronary heart disease: consensus statement of the European Atherosclerosis Society. European heart journal. 2013;34:3478-3490a.
2. World Health Organization. Familial hypercholesterolemia—report of a second WHO Consultation. Geneva, Switzerland: World Health Organization, 1999. (WHO publication no. WHO/HGN/FH/CONS/99.2).
